# Heads up on concussion: Aboriginal and Torres Strait Islander peoples' knowledge and understanding of mild traumatic brain injury

**DOI:** 10.1002/hpja.892

**Published:** 2024-07-11

**Authors:** Kahlia McCausland, Elizabeth Thomas, Jonathan Bullen, Trish Hill‐Wall, Richard Norman, Gill Cowen

**Affiliations:** ^1^ Collaboration for Evidence, Research and Impact in Public Health, School of Population Health Curtin University Perth Western Australia Australia; ^2^ Centre for Clinical Research Excellence Curtin University Perth Western Australia Australia; ^3^ Division of Surgery, Faculty of Health and Medical Sciences The University of Western Australia Perth Western Australia Australia; ^4^ Curtin enABle Institute Curtin University Perth Western Australia Australia; ^5^ Telethon Kids Institute Perth Western Australia Australia; ^6^ Office of the Deputy Vice‐Chancellor Curtin University Perth Western Australia Australia; ^7^ Moorditj Yorga Scholarship Program Curtin University Perth Western Australia Australia; ^8^ Curtin Medical School Curtin University Perth Western Australia Australia; ^9^ School of Population Health Curtin University Perth Western Australia Australia; ^10^ Curtin Health Innovation Research Institute, Curtin University Perth Western Australia Australia

**Keywords:** Australian Aboriginal and Torres Strait Islander peoples, Australian Aboriginal peoples, brain concussion, Indigenous Australians, mild traumatic brain injury, public health, Torres Strait Islander peoples

## Abstract

**Issue Addressed:**

Concussion awareness and knowledge among Aboriginal and Torres Strait Islander peoples residing in Perth, Western Australia and factors preventing presentation at a health service for assessment after such an injury.

**Methods:**

Qualitative study with participants aged between 18 and 65 years. Recruitment was by Facebook advertising and snowball sampling. A semi‐structured topic yarning guide was used to guide conversations through 1:1, multi‐person or group yarns. Yarns were audio‐recorded, transcribed and thematically analysed.

**Results:**

Twenty‐four participants were recruited. A good knowledge of modes of concussion injury was identified in these participants. However, they identified difficulty differentiating this injury from other injuries or medical conditions. Multiple factors contributed to a reluctance to seek assessment and further management of a potential concussion. Multiple strategies to enhance education and presentation for assessment were suggested by participants.

**Conclusions:**

Aboriginal and Torres Strait Islander‐owned and led concussion education is the first step in enhancing understanding of this condition. Education must be coupled with improvements in the cultural safety of healthcare services, as without this, patients will continue to fail to present for assessment and management.

**So What?:**

It is recommended that concussion education focuses on the differentiation of concussion as a diagnosis from other injuries. Information regarding where and when to seek medical assessment is recommended, and this must be in a culturally safe environment. Typical recovery and potential sequelae must be explored, in programs led and devised by Aboriginal and Torres Strait Islander peoples engaged with the community for which the education is proposed.

## 1 BACKGROUND

Mild traumatic brain injury (mTBI), or concussion, is caused by a direct blow or transmitted force to the head^1^ and presents differently for different individuals.^2^ There are no universal criteria for diagnosing concussion, but common symptoms include headache, confusion, fogginess, mood disturbance, memory loss, fatigue or balance and vision problems.^3^ Most people will recover from a concussion^4^ with a gradual and paced return to mental and physical activity.^5^ Still, recovery times vary significantly between individuals, and some people can experience long‐term^6^ and function‐altering effects after their injury.^7^

The spectrum of presenting symptoms can make diagnosing a concussion difficult. Some health professionals use tools such as the Post Concussion Symptom Scale, Sports Concussion Assessment Tool, Rivermead Post Concussion Symptoms Questionnaire and Brain Injury Screening Tool to assist their assessment.^8^, ^9^, ^10^ There is limited knowledge among the wider community on identifying a potential concussion and the need to seek medical assessment.^11^ This is particularly true in lower socioeconomic demographics, where there is a tendency for health literacy to be lower in general.^12^

Previous research has shown that Indigenous populations are particularly vulnerable to all types of traumatic brain injury (TBI) and that knowledge on the identification of concussion is poor in First Nations communities.^13^, ^14^ Research also shows that in some Indigenous athletes, there is a reluctance to report head injuries, for fear of being excluded from play.^15^ In Aboriginal and Torres Strait Islander peoples, presentation to health services for head injury is further complicated by an inherent mistrust of institutional care.^16^, ^17^, ^18^ Australia is marked by a suppressive history of Aboriginal and Torres Strait Islander peoples, leading to enduring intergenerational physical and psychological impacts^19^ that negatively influence tendencies to seek healthcare and add to the tendency of hospital avoidance.^20^

In this study, we investigated the knowledge of concussion among Aboriginal and Torres Strait Islander peoples residing in Western Australia (WA), with the aim of better understanding the need for improved education and service provision for this vulnerable group.

## 2 METHODS

### 2.1 Research team

The research team consisted of three Aboriginal Australians and three non‐Aboriginal investigators. JB is a Wardandi Noongar man, whose research focuses on expanding the lens through which Aboriginal health and well‐being are viewed, understood and promoted. ET is from the Yamaji region and works as a Research Fellow coordinating research across rural and remote WA. TH‐W is a proud Noongar Elder with extensive family connections in Yamatji, Wongi and other regions of WA. GC is a rural‐trained General Practitioner with a special interest in sports medicine. RN is a Health Economist whose research focuses on health outcomes and evidence‐based policy. KM is a Research Fellow who has experience conducting research with marginalised populations, which has provided her with insight (although not personal experience) into the social and structural challenges these groups have and continue to face.

### 2.2 Context and governance

This research is the second phase of a broader research project. Phase 1 sampled Aboriginal Australians with high health literacy.^18^ The research team developed the yarning topic guide that was used in Phase 1 and adapted for Phase 2. Research team member and Aboriginal Elder TH‐W was employed as a research assistant (Phase 1) and mentored the non‐Indigenous lead researcher (GC), ensuring that Indigenous standpoints and research methodologies were considered and applied appropriately. Although Aboriginal team members were not directly involved in data collection and analysis in Phase 2, all members significantly contributed to project conceptualisation, development of data collection instruments and processes, interpretation of the data and knowledge sharing and education of non‐Indigenous researchers.

### 2.3 Methodology

This qualitative research study adopted a social constructionist lens^21^ which emphasises the significance of culture and context in shaping our interpretations and comprehension. It asserts that knowledge is a product shaped by society and that truth relies on agreement within a specific community rather than having a universal definition. The adoption of social constructivism facilitates an exploration of the meanings Indigenous Australians ascribe to their experience and acknowledges the influence of their worldviews. The reporting of this study is guided by the Standards for Reporting Qualitative Research.^22^

### 2.4 Sampling and recruitment

Eligible participants were required to identify as Aboriginal and/or Torres Strait Islander, aged between 18 and 65 years and currently staying or residing in Perth, WA. A total of 24 participants were recruited through purposive sampling, 19 through Facebook advertising and 5 through snowball sampling.

Facebook's advertising service was utilised to broadly target Facebook users, aged 18–65+ years who had their location set within a 70‐km radius of Perth, WA (as Facebook does not allow targeting based on ethnicity or race). AU$1083 was spent on paid advertising over 9 weeks (18 March to 29 May 2022). During this period, the recruitment post reached 70 529 individuals and resulted in 1264 link clicks, equalling AU$0.86 per link click (Appendix 1). The average cost in advertising fees per eligible participant interviewed equated to AU$60.

The Facebook recruitment advertisement directed people to register their interest in participating in the study via a QR code or URL, both of which directed them to an expression of interest survey that asked the following screening questions:

1. Age
2. Are you an Indigenous Australian currently living in Perth?
3. Are you trained in, currently training in, or working in healthcare or first responder services?
4. Are you working in an academic position related to health care or first responder services?

People who answered ‘no’ to question 2 or ‘yes’ to either questions 3 or 4 were not eligible to participate. People left their preferred contact information so they could be contacted by the research officer (KM). Data were collected using REDCap (Research Electronic Data Capture) hosted at Curtin University.^23^ REDCap is a secure, web‐based software platform designed to support data capture for research studies.^24^ A total of 137 people expressed interest in participating via REDCap, of which 15 (11%) were deemed ineligible, 96 were lost to follow‐up (70%), 7 declined upon follow‐up (5%) and 19 were interviewed (14%). Two participants were related to one another and recruited their family and friends (*n* = 5).

### 2.5 Data collection

A semi‐structured topic yarning guide (Appendix 2) was adapted from our prior research.^18^ Due to the COVID‐19 pandemic, participants were given the option of several participation methods. Eleven participants participated in a 1:1 yarn, six participated in a multi‐person yarn, and one group yarn was conducted (*n* = 7). Research topic yarning was preceded with a social yarn in all cases.^25^ Eighteen participants participated face‐to‐face, 5 participated by phone and 1 online. Data were collected by the chief investigator (GC) and research officer (KM).

Following the provision of participant's consent, they were asked to complete a demographic survey (Appendix 3) before moving into the yarn, whereby participants were encouraged to share their (and their mobs') experience of concussion and their awareness and understanding of the term. During the exchange of knowledge between the facilitator and participant/s, incidental concussion education was provided by the facilitator. Yarns were audio recorded with participant consent.

Participants were remunerated for their time and provided with a $50 gift card for a supermarket chain (Coles). Refreshments were also provided to face‐to‐face participants.

### 2.6 Data analysis

Audio recordings were transcribed by a professional transcription service and then reviewed for accuracy by three volunteer medical students and the research officer, with any inaccuracies corrected. De‐identified transcripts were imported into NVivo^26^ to facilitate data organisation and linkage. Reflexive thematic analysis, the six‐phase process set out by Braun and Clarke,^27^ was used to engage with the data.

Two researchers (KM and GC) read all transcripts to facilitate data familiarisation and the generation of initial codes. One of the researchers (KM) then inductively coded all transcripts to generate draft codes and themes relevant to the experiences of participants that have influenced their knowledge and understanding of concussion. The other researcher (GC) also coded all transcripts; however, they specifically sought to identify instances where participants identified injury events, symptoms, signs and patient outcomes that were in keeping with a diagnosis of concussion, and those where an alternate diagnosis such as a moderate to severe TBI was discussed. Upon completion of coding, researchers came together to discuss the draft codes and themes. One researcher (KM) refined coding based on the discussion and drafted the first narrative summary. The draft summary was reviewed (GC) and further refined (KM); themes were collapsed and new themes were generated. The preliminary analysis report with supporting participant extracts was reviewed by the broader research team resulting in further theme and subtheme refinement.

### 2.7 Ethical considerations

This study was approved by the Derbarl Yerrigan Health Service Aboriginal Corporation Research and Development Sub‐Committee. Derbarl Yerrigan's process for reviewing research proposals has been designed in consultation with the WA Aboriginal Health Ethics Committee (WAAHEC) and appraises projects using the Guidelines for Ethical Research in Australian Indigenous Studies.^28^ Ethics approval was obtained from the WAAHEC (HREC1012) and reciprocal ethics approval from Curtin University (HRE2020‐0690). Findings from this research will be presented back to the WAAHEC, Derbarl Yerrigan, Connectivity Traumatic Brain Injury Australia^29^ and study participants.

## 3 RESULTS

A summary of participants' demographic characteristics is presented in Table 1. Of the 24 participants, the majority identified as Aboriginal (*n* = 22, 92%) and female (*n* = 16, 66%), and were aged between 25 and 45 years old (*n* = 12, 50%). Most participants had completed at least Year 10 (*n* = 18, 75%), with others going on to complete vocational education and training (*n* = 10, 42%) and higher education qualifications (*n* = 5, 21%). Over half of the participants were employed (*n* = 13, 54%) and reported caring for immediate and/or extended family (*n* = 14, 58%). Interviews lasted on average 68 min (range 35–96 min).

**TABLE 1:** Participant demographic characteristics.

Characteristic	Number	Percent
Cultural identity		
Aboriginal	22	92
Torres Strait Islander	1	4
Missing data	1	‐
Age		
18–25 years	7	29
25–45 years	12	50
>45 years	5	21
Gender identity		
Male	8	33
Female	16	66
Education		
Year 10	18	75
Year 12	9	38
Vocational education and training	10	42
Higher education	5	21
Student		
Yes	1	4
Employed		
Yes	13	54
Carers responsibilities^a^		
Yes	14	58

^a^ Carers responsibilities denote participants who are responsible for the management and support of children, parents and/or extended family.

## 4 FINDINGS

Six predominant themes were identified. A narrative summary, with supporting quotes, is presented. Quotes have been attributed a pseudonym to protect the participant's identity. In the case of the group interview, it was not possible to attribute each participant's age.

### 4.1 Good understanding of how concussion injury can occur

Participants mostly demonstrated a good understanding of how concussion injury can occur, drawing on their personal experiences of playing and watching sports, generally Australian rules football, netball and rugby.

We grew up around sports and footy is big in our family. So not every week, but there'd be out in country footy every now and then, somebody would get hit hard, whether it was a bump or whether it was the head hitting the ground. So we saw that quite a bit. So we was aware of concussion … Concussion is what you call it when you get hit to the head. [Bouddi, M, 41]

Other modes of concussion injury recalled included interpersonal violence (including intimate partner violence), falls and road traffic accidents.

… there is obviously a lot of domestic abuse within the Aboriginal community where it's more than often women who‐ like even in my own situation, he never punched me as such in the face or head, but he would push me and shove me and so‐ Yeah, definitely from my body and I never really, you know, you'd have a headache, you'd feel sore … [Medika, F, 50]

The impact of alcohol was also recognised by one participant as increasing concussion injury risk:

… they're drunk, they're not really steady on their feet when they're drunk and they might fall over and hit their head. But then also, alcohol can cause aggression in other people and you can be put in a situation where you end up getting punched in the head. [Kirra, F, 44]

### 4.2 Diagnosis is complex and difficult to differentiate

It was evident that participants found it difficult to differentiate concussion from other conditions. Participants described concussion symptoms and signs including light‐headedness, dizziness, vomiting, lack of spatial awareness, headaches, migraines, sensitivity to light and noise, memory and sleep disturbance, poor balance or coordination, blurred vision, nausea, confusion, vertigo, fatigue, whiplash, grogginess, seeing stars, unable to concentrate, disorientation, not talkative/quiet, neck pain, being slow to get up and mental health issues, but also described such presentations being experienced or observed with other diagnoses. Some participants incorrectly described a concussion as requiring a loss of consciousness or being ‘knocked out’. Other signs and symptoms participants reported that are not typically concussion‐related included ringing in the ears, pressure in the skull requiring surgery, fainting, dyslexia, increased thirst, trouble breathing, paleness, drop in body temperature, difficulty talking, dilated pupils and coma:

… he didn't wake up for three months. [Karen, F, 46–65]

Participants discussed persistent concussion symptoms of memory loss, mental health challenges, mood and behaviour disturbance, recurring headaches and migraines, deterioration of vision, blurry vision, poor exercise tolerance, agitation, light and noise sensitivity, fatigue, poor concentration, and sleep disturbance. Some participants expressed it was difficult to know whether to attribute symptoms to the injury or whether symptoms were coincidental.

Yeah, just he's [person who had the injury] not as methodical anymore. He's not as thoughtful. … And still to this day, we can't compromise with him. It's like trying to deal with a three‐year‐old. … It's like he switches into a different mode. But I don't know if that's because of the knock, but it did change him … [Kirra, F, 44]

A couple of participants identified the potential for persisting cognitive issues to stem from repeated ‘knocks’ to the head but also reported that drug and alcohol misuse confounded such situations.

Because my memory's been quite bad. I've grown up with the same two best mates, and they recall stories and I can't really recall them … I've drank a fair bit of booze and stuff in my time, so maybe it's just from that. [Nullah, M, 32]

One participant identified increased susceptibility to seizures because of recurrent head injuries and two participants reported the development of epilepsy. This suggests a lack of clarity between a diagnosis of concussion and moderate/severe TBI.

### 4.3 Concussion awareness and knowledge are gained from various sources

Participants described obtaining concussion awareness and knowledge from watching community and televised sporting events, other media, lived experience and word‐of‐mouth. Typically, concussion awareness was acquired by witnessing how seriously potential concussions were managed at community and televised sporting events.

I think now through football we're starting to see that it's being taken more seriously. [Rianna, F, 53]

… watching MMA [mixed martial arts] or UFC [ultimate fighting championship] and seeing people get KO'd [knocked out] and then they get up … And they don't even recall what happened. … they'll do basic tests on them like, ‘Do you know what day it is?’ And they were like, ‘Oh, I'm not too sure what today is.’ And it showed the severity anyway. [Jarrah, M, 35]

Further, concussion awareness and knowledge were obtained from the media.

I've mostly only heard of it [concussion] through the media, I suppose, through sports and that, and I think it's only come to light really in the last few years … [Marli, F, 36]

Just the last few years, just really watching footy and watching all the players that have retired from footy or even quit because they can't play anymore. … so I think I was like, oh, ok. Anytime I get a head knock where I'm not a hundred percent comfortable about it, especially in my old age, in footy terms, getting towards the end of my career [I will go and get it checked out] … [Josh, M, 32]

Personal experiences of concussion and the passing of intergenerational knowledge also played a role in participants' concussion awareness and knowledge.

Well, I grew up with a lot of abuse so getting head knocks was a daily occurrence. And then we did talk about it. We knew about concussions. I guess Mother or Nan would like, ‘Oh … Don't let them go to sleep. Make sure that they're not going to be passing out or vomiting’ … [Yindi, F, 32]

Some participants recalled they had received brief concussion education through first aid courses; however, when probed by the facilitator, their information pertained to the management of someone who appears unconscious, not specifically to concussion.

### 4.4 Concussion may not be considered a serious injury

Some participants shared the perception that potential concussions are not being taken seriously.

… we don't take it [head injury] seriously enough … We dust that off. [Kylie, F, 26]

In adults, minimisation of injury or symptoms was commonly reported.

… if I was playing sport and I copped a knock to the head … I wouldn't actually seek medical attention. [Kirra, F, 44]

[Would I seek medical attention] For myself, no, probably not, unless I had a gash or something or I had knocked myself out. [Marli, F, 36]

Even participants with prior personal experience of concussion detailed occurrences when they had minimised their injury and passed it off as ‘just a head knock’:

I did a rough tackle on someone else, but I hurt myself in the process. I didn't think I had a concussion … but I felt like my head hurt more than it should have. … I didn't get referred [to a doctor]. I was like, it's just a head knock. [Jedda, F, 22]

Some older participants recounting their junior sporting days referred to a culture within sporting clubs, whereby personal injury was unimportant compared with commitment to the team.

… you took a knock to the head or landed wrong and it was, man up, get up. And there was nothing about, oh God, you've shaken your little brain around a bit. [Rianna, F, 53]

A similar culture and commitment to the team were also reported among younger participants who recalled times when they continued to play out a game after a potentially concussive event.

I told the coach, I was like, ‘No, no, no, I was just laying down there because I was sore, I wasn't knocked out.’ So he let me go back on and then I run out there and I run for about two seconds and then just started throwing up … [Allira, F, 21]

### 4.5 Barriers to seeking healthcare

Participants identified several key barriers to seeking healthcare, including a lack of cultural safety within healthcare services; concepts of shame and pride; financial and logistical challenges; and home and carer's duties.

#### 4.5.1 Mistrust of government agencies

Participants documented experiences of prejudice, stigma, racism, and discrimination within healthcare services. These experiences reinforced their mistrust of government agencies.

It [racial discrimination] happens quite often and myself I'm fairer, so I've had the privilege where people can't immediately tell I'm Aboriginal, but I have been in hospitals with relatives who are darker skinned, and often they won't go, they'll say, ‘Oh, you take me’. Because they know I'm fairer skin, they feel like they'll get the attention that they need. [Medika, F, 50]

[Aboriginal people are] Not taken seriously. I've heard people [healthcare professionals] like, ‘Oh, they're just probably drunk’ … And the look is like a look of disgust. It's like, you disgust me being here. [Jarrah, M, 35]

Fear was especially heightened in cases where someone had been involved in a fight or the injury occurred secondary to family violence with concern that the authorities would be called upon presentation at a healthcare service.

Most participants were more likely to present children for assessment at a healthcare service than themselves: *‘*For the kids, yes’. [Marli, F, 36] However, due to Australia's involvement in Indigenous child removal, a deep mistrust of government agencies and the ‘system’ was expressed when children were involved, with real fear conveyed that their children would be taken away.

… if there was a couple fighting and stuff, then there's a whole case of what's going to happen with DCP [Department of Child Protection] and children, and I'm too scared to report it, because my kids might get taken away. [Jarrah, M, 35]

… if you show up at any medical place in distress, especially being Indigenous, they're writing notes on you. … Those reports go straight to the department. That makes me think, no, even if I had a serious concussion, the last place I would want to go, me and my kids, would be to the hospital. Because if I was to lose consciousness or something, what would they do to my kids? … When you compare yourself as a parent, your medical needs are just way on the bottom compared to a possible department engagement. [Kylie, F, 26]

Participants identified that healthcare services (particularly hospitals) were perceived, especially by those from the Stolen Generation, as oppressive institutions that can represent separation from family.

… we haven't gone past the stolen generation, there's still the intergenerational trauma from that. … Like that's still a real threat. … but you don't want your children taken away and you don't necessarily feel like you're going to get the support either. [Medika, F, 50]

The paucity of Aboriginal and Torres Strait Islander people working within healthcare services and its effect on presentation for medical assessment was also discussed.

I think that we should have more Aboriginal health workers, even if it's like just Aboriginal liaison, peer support workers … I think a lot of Aboriginal people have that mindset of it's run by the white man so I don't want to [go]. [Allira, F, 21]

In particular, the lack of male Indigenous healthcare professionals was reported to further compound some men's avoidance of healthcare services, as some Aboriginal and Torres Strait Islander men often prefer to discuss their business with another man.

And I know that a lot of … Guys would prefer to talk to another guy. [Nullah, M, 32]

As a result of poor experiences with healthcare services, some felt safer presenting to an Aboriginal medical service.

So I personally only go to [service name redacted] … I feel like it's just a safer space than going to a really clinical hospital or a GP [general practitioner]. I feel more safer because I've gone [there] my whole life. [Jedda, F, 22]

Sometimes … they look up your record and they see you're Aboriginal, the care factor may go up or may go down. If the person that I was assisting needed treatment and they were Indigenous, I would prefer to take them to an Aboriginal medical center … [Rianna, F, 53]

However, presenting to an Aboriginal medical service was sometimes coupled with other challenges such as a perceived lack of privacy.

… some people won't go to an Aboriginal medical service because they've got family working there and their business is going to get sprayed all over town. So they'll happily go and see the white fellas. But they might not be treated like they would at the [Aboriginal medical] service, but at least they know their business stays their business. [Rianna, F, 53]

#### 4.5.2 Shame and pride

Shame, attached to other factors including alcohol intoxication, illiteracy and family violence; and pride, including concepts of masculine pride and intergenerational role modelling, inhibited participants from seeking healthcare.

… they've hit their head on the gutter and they don't want to go to the hospital and tell that they've been drunk and fallen. [Kirra, F, 44]

… with my mum, she wouldn't go to the hospital because she couldn't read or write, and they do usually hand you a form. [Kirra, F, 44]

… like if they have been in domestic violence, they might be trying to protect their family or their partner from any repercussions, like legally from that situation. … even though you are the victim somehow it's a shame that's associated with it. [Medika, F, 50]

That's sookylala behaviour to go and do that [seek medical assistance]. … our Indigenous fellas are still quite manly. You don't do that. … still have their rep[utation] to protect and still got to be a man. [Rianna, F, 53]

#### 4.5.3 Practical challenges

Two participants recognised the cost of healthcare as a barrier to seeking help.

… then there's been times where I've needed … urgent medical assistance and I couldn't afford the ambulance … [Kylie, F, 26]

The perceived prolonged wait time at hospitals was a deterrent for participants who would rather opt to not seek medical assistance at all or to wait, sometimes several weeks, to see a general practitioner.

Mum took me once [to the hospital], but I kind of refused to go in. She's like, ‘Tarni, you need to get checked’. And I'm like, ‘No, I'm good, let's just go home’. I'm very impatient … knowing that I've got to sit there for hours … [Tarni, F, 34]

Two female participants identified that home and carer's duties often preclude them from seeking personal medical care.

What would I do with my kids when I have to present at the hospital, and I don't have any family or friends around me to help? [Kylie, F, 26]

### 4.6 Opportunities for concussion information sharing

Participants identified opportunities for concussion information sharing in terms of need, specific content and strategies, and the integration of concussion information sharing within existing services.

#### 4.6.1 A need for concussion information sharing

Participants identified a lack of knowledge and understanding about concussion within Aboriginal and Torres Strait Islander communities and through descriptions of how a potentially concussive event might be managed.

I think we have minimal knowledge on it [concussion] … I've never been told the seriousness of a hit to the head … [Kylie, F, 26]

And I think some people … They're not really educated about concussion and the effects. So I think they just think it's a head knock so they won't go to a doctor. [Jedda, F, 22]

I'd actually let it go longer before I would seek medical attention, which doesn't makes a lot of sense. … I guess in my own way, I'd have to see more evidence of myself being affected by it before I would get medical attention. [Kirra, F, 44]

Despite sport being a key avenue participants identified as increasing their concussion awareness and knowledge, there was little formal concussion education by community sporting clubs reported.

Not until I'd been concussed for probably like the sixth time … then they [sporting club] started bringing it up, trying to just educate a little bit on be[ing] careful, not telling us what happens [to the brain with a concussion], but just be careful, your head's the most important part. [Allira, F, 21]

#### 4.6.2 Information sharing opportunity—Content and strategies

As a result of the incidental concussion education provided by facilitators during interviews, participants identified information that they perceived as useful to be widely shared, including that concussion can occur from a transmitted force to the head, not just a direct blow; and advice around initial concussion assessment, management and prevention (such as falls prevention in the elderly).

Participants felt it was most important to provide concussion information to young people, especially in regional and remote communities, as young people are likely to provide secondary education to their parents and other family members.

… start educating the youth, because the youth are the ones who are going to be the adults, and the more knowledge that they have around health in general, and their own health … going to … the grassroots footy club, the youths, the sporting clubs where it happens, into the home, into the communities where we have lots of the children. [Bouddi, M, 41]

It was also suggested to share concussion information with Aboriginal and Torres Strait Islander communities ‘through the channels that they're already on’ [Nullah, M, 32], which included the use of interactive apps; social media; workshops and training sessions; mainstream promotion consisting of pamphlets, posters, TV advertising and streaming services; and weaving concussion education into the narrative of children's TV shows.

Yeah, probably by putting on TV ads, an example, like those quit smoking ones, because they're presented quite well. … It's engaging and we started to love them, because that's our people. Do we know anyone that is suffering from a bad knock, concussions and stuff? So, we want to look at them. That means we're taking that information without even knowing, that useful information. [Kylie, F, 26]

Furthermore, the use of catchy slogans; promotional merchandise (e.g., t‐shirts); incentives; and an ‘ambassador for head knocks’ such as an influential well‐known person who has personal experience of concussion, or an Aboriginal and/or Torres Strait Islander educator or elder were proposed.

The deadly health checkups, they give away a free shirt. … but we love getting a free shirt with the pattern on it. Everyone gets it from their local area. … the local artist will design a shirt. [Kirra, F, 44]

#### 4.6.3 Information sharing opportunity—Integration within existing services

Participants proposed that concussion information sharing be integrated within existing services such as Centrelink, Aboriginal medical services, general practitioners, education courses (e.g., Aboriginal healthcare and mental health certificates), yarning and community groups, outreach services, sporting clubs and family violence counselling. One participant recommended a ‘neck up checkup’ with concussion information sharing included within mental and general health assessments:

So mental health and your brain activity and all of that is all important, even your emotional wellbeing, your spiritual as well, because that really is not just in you, it's also in your mind. … [One project] we do that's national is the deadly health checkups. … [But] there's no neck‐up checkup. It's all from the neck down. [Kirra, F, 44]

## 5 DISCUSSION

Limited studies explore Indigenous peoples' understanding of TBI. This study explores concussion knowledge and understanding among Aboriginal and Torres Strait Islander peoples who do not have a background in health or first response, providing additional information to that provided by Hill‐Wall et al.^18^ in a health and first response cohort.

Whilst our sample, residing in Perth, WA, had a good understanding of modes of concussion injury, we identified knowledge gaps that provide further opportunities for concussion awareness and education strategies, and primary and secondary prevention. In this study, we identified limitations in awareness of the pathophysiology of concussion and subsequent symptom minimisation, which is likely to reinforce the assumption that concussion is not a ‘significant’ injury. In addition, the complexity of diagnosis due to the overlap of symptoms and signs with other co‐existing conditions such as whiplash, intoxication and non‐fatal strangulation may also contribute to the misattribution of concussion symptoms and signs.

We also identified a lack of clarity regarding the difference between concussion and other types of TBI or head injury. This finding replicates previous research in this area^18^ and is consistent with other literature identifying misconceptions about TBI.^30^ There is a risk that patients may feel inappropriately reassured after the exclusion of a certain diagnosis. For example, having an intracranial bleed excluded after a head injury then leaving the emergency department without considered an alternative diagnosis of concussion and returning immediately to high‐risk activities.

Such lack of clarity, and risk of poor outcome, are further confounded by problems highlighted by participants when considering whether to present for assessment and management at all. These include a lack of culturally appropriate facilities or staff, as well as the deep‐seated mistrust of healthcare and government systems^31^, ^32^ that has resulted from previous mistreatment of Indigenous peoples.^33^, ^34^ Data collected in this study highlights how personal experiences influence presentations and the influence of shame on interaction with medical services and medical professionals.^35^, ^36^, ^37^ Until Aboriginal and Torres Strait Islander peoples feel safe to present to such services, long‐term outcomes after concussion are unlikely to be optimised. This is of particular concern given the associations reported between mTBI and neurodegenerative diseases.^38^, ^39^, ^40^, ^41^

A key finding in this study is the identification of areas where further opportunities for incidental or structured concussion education can occur. Our results indicate that concussion knowledge is acquired from community and televised sports, and other media including social media and through lived experience and intergenerational knowledge transfer. As such, a multifaceted approach to concussion awareness is recommended. This is further supported by previous research that supports multisector primary prevention strategies.^42^, ^43^, ^44^, ^45^, ^46^, ^47^ Furthermore, any such strategies must be strength‐based and driven by Aboriginal and Torres Strait Islander peoples, to ensure cultural security, feasibility and appropriateness for those being targeted, and to ensure the approaches taken do not replicate deficit models of research and thus research translation that has been so dominant.^48^

Participants provided clear support for processes that involved education and prevention strategies driven by sports of all kinds. Such education would build upon pre‐existing community networks and leverage pre‐existing community relationships and strengths. Aboriginal and Torres Strait Islander ambassadors for concussion are suggested to foster community engagement with Indigenous sportsmen and women championing such engagement. Professional sports are big business, and it is suggested that revenue from televised sports events and competitions may be in part diverted into televised concussion educational pieces with brief information segments sitting alongside game commentary and advertising. Given the diversity of modes of concussion injury, the scope for such advertising could be far‐reaching including not just the traditional contact team sports.

There was also advocacy for the use of existing health promotion strategies or processes that were felt to already be successful, such as fall prevention programs, and the provision of incentives to participate, such as the Deadly Choices^49^ health promotion initiative where participants receive a specially designed t‐shirt. The suggestion by one participant of a ‘neck‐up checkup’ was of particular interest, with the suggestion that it was included in already available physical health checks and specifically included additional education and screening. The idea of leveraging off successful pre‐existing campaigns is a potential way to access already engaged Aboriginal and Torres Strait Islander peoples; however, caution must be exercised to ensure such pre‐existing services are not overburdened with additional responsibility, asked to provide services in which they have insufficient training and/or knowledge, or are not perceived as useful or safe by their community.

It was reported that concussion education is passed down through generations, but there was support and scope for the education of children and adolescents who could pass information to their parents, uncles, aunties and other family members, as well as the next generation. This approach would allow for novel education strategies through popular social media platforms and indeed popular children's television programs.^50^ Television and other media advertising were also identified as a possible portal for concussion education, and previous campaigns such as those for smoking cessation^51^ were highlighted by participants as useful for spreading information in a culturally appropriate and relatable way. Whilst such strategies would have the benefit of a wide reach, caution must be taken to ensure consistent, reliable best practice information is provided, and content expert opinion is recommended.

### 5.1 Limitations

Social media was utilised to broaden the reach of participant recruitment.^52^ Of the total 70 529 Facebook users who saw our recruitment advertisement, 137 people expressed interest in participating and 19 people were interviewed as a direct result of this recruitment strategy, resulting in a recruitment cost of AU$60 per person. Although Facebook allows advertisers to target some specific audience characteristics such as gender, age and location, individuals cannot be targeted by ethnicity or race, which limited the specificity of our recruitment strategy.

Data was collected by non‐Indigenous researchers which may have impacted the richness of the data collected. Furthermore, formal data analysis was undertaken by non‐Indigenous researchers. We acknowledge the divergence in cultural positioning between non‐Indigenous individuals and the potential influence this could have had on the development of research findings. Despite the absence of direct participation from an Indigenous individual in the data collection or formal analysis stages, input and questioning by Aboriginal co‐investigators encouraged the consideration of alternative viewpoints, interpretations, and concerns. We have confidence that the involvement and supervision of our Aboriginal colleagues have helped mitigate these acknowledged limitations. Another limitation is that data was only collected from Aboriginal and Torres Strait Islander peoples living in the Perth region of WA. Further research is required to identify if opinions and concussion knowledge are similar in other areas of Australia, including regional, rural, and remote areas.

## 6 CONCLUSION

This study has identified that despite good knowledge of modes of concussion injury, there remain some knowledge gaps and opportunities for further education. A difficulty in differentiating a concussion from moderate or severe TBI was identified. Reasons for failing to present for assessment were multifactorial, but inter‐generational trauma and lack of culturally appropriate care played a significant role in this reluctance. Participants also reported symptom minimisation due to pride or a feeling of responsibility to return to play. Participants provided investigators with multiple potential strategies to enhance concussion knowledge in their communities, highlighting the need for community‐led education strategies and Aboriginal and Torres Strait Islander ownership of such projects.

## CONFLICT OF INTEREST STATEMENT

Dr Gill Cowen is Chair of the Western Australian Concussion Network, a member of Connectivity Traumatic Brain Injury Australia and sits on the Community and Regional Football Committee of the Western Australian Football Commission.

## Supporting information


**Appendix 1.** Facebook recruitment advertisement performance metrics.

**Appendix 2.** Semi‐structured yarning topic guide.

**Appendix 3.** Demographic survey.

## Data Availability

Research data are not shared.
