# Supplementary material for: Heads up on concussion: Aboriginal and Torres Strait Islander peoples' knowledge and understanding of mild traumatic brain injury
Source: Health Promot J Austr. 2024 Jul 11;36(1):e892. doi: 10.1002/hpja.892 (PMC11729264; doi:10.1002/hpja.892)
Supplement: Supplementary file 1 — Appendix 1. Facebook recruitment advertisement performance metrics. [file HPJA-36-0-s003.docx]

**Appendix 1. Facebook recruitment advertisement performance metrics**

| **Metric** | **Performance** |
| --- | --- |
| Reach^[[1]](#footnote-1)^ | 70,529 |
| Post engagement^[[2]](#footnote-2)^ | 1,574 |
| Link clicks^[[3]](#footnote-3)^ | 1,264 |
| Cost per link click^[[4]](#footnote-4)^ | $0.86 |
| Post shares | 128 |
| Post reactions | 108 |
| Post comments | 51 |
| Post saves | 23 |

1. Reach: The number of people who saw the ad at least once. [↑](#footnote-ref-1)
2. Engagement: The total number of actions people took involving the post. This includes liking/reacting, commenting, sharing, clicking the link, etc. [↑](#footnote-ref-2)
3. Link clicks: The number of clicks on links within the ad that led to destinations or experiences, on or off Facebook. [↑](#footnote-ref-3)
4. Cost per link click: This metric is calculated as the total amount spent divided by the number of link clicks. [↑](#footnote-ref-4)
