# Supplementary material for: Heads up on concussion: Aboriginal and Torres Strait Islander peoples' knowledge and understanding of mild traumatic brain injury
Source: Health Promot J Austr. 2024 Jul 11;36(1):e892. doi: 10.1002/hpja.892 (PMC11729264; doi:10.1002/hpja.892)
Supplement: Supplementary file 2 — Appendix 2. Semi‐structured yarning topic guide. [file HPJA-36-0-s001.docx]

**Appendix 2. Semi-structured yarning topic guide**
