# Supplementary material for: Heads up on concussion: Aboriginal and Torres Strait Islander peoples' knowledge and understanding of mild traumatic brain injury
Source: Health Promot J Austr. 2024 Jul 11;36(1):e892. doi: 10.1002/hpja.892 (PMC11729264; doi:10.1002/hpja.892)
Supplement: Supplementary file 3 — Appendix 3. Demographic survey. [file HPJA-36-0-s002.docx]

**Appendix 3. Demographic survey**

1. **Age: ­­­­­­­­­­­­­­­_____________________________________________________________________**

**OR select from the groups below:**

18-24 years old

25-45 years old

46-65 years old

1. **Gender:**

Male

Female

Non-binary

1. **What country do you identify with? ___________________________________________**
2. **What suburb do you live? Or you can write your postcode: ________________________**
3. **Did you finish Year 10?** Yes  No
4. **Did you finish Year 12?** Yes  No
5. **Are you currently studying?** Yes  No

**If yes, what course are you studying? _____________________________________________**

1. **Do you have a certificate, diploma or university degree?** Yes  No

**If yes, what qualification is this? ­­­­­__________________________________________________**

**_____________________________________________________________________________**

1. **Are you currently working?** Yes  No

**If yes, what do you do for work? __________________________________________________**

**_____________________________________________________________________________**

1. **Do you have carers responsibilities (e.g. looking after children, parents, extended family)?**

Yes  No

**If yes, who do you care for? _____________________________________________________**
